# Dosimetric comparison of high dose rate brachytherapy and intensity-modulated radiation therapy for cervical carcinoma

**DOI:** 10.4103/0971-6203.79687

**Published:** 2011

**Authors:** B. Shwetha, M. Ravikumar, Siddanna R. Palled, Sanjay S. Supe, S. Sathiyan

**Affiliations:** Department of Radiation Physics, Kidwai Memorial Institute of Oncology, Bangalore, India; 1 Department of Radiotherapy, Kidwai Memorial Institute of Oncology, Bangalore, India

**Keywords:** Cervical carcinoma, high dose rate, intensity-modulated radiotherapy, intracavitary brachytherapy

## Abstract

Intracavitary brachytherapy is an integral part of radiotherapy for locally advanced gynecologic malignancies. A dosimetric intercomparison of high dose rate intracavitary brachytherapy (HDR_BT) and intensity-modulated radiotherapy in cervical carcinoma has been made in the present study. CT scan images of 10 patients treated with HDR_BT were used for this study. A sliding-window IMRT (IMRT_SW) and step-and-shoot IMRT plans were generated using 6-MV X-rays. The cumulative dose volume histograms of target, bladder, rectum and normal tissue were analyzed for both techniques and dose distributions were compared. It was seen that the pear-shaped dose distribution characteristic of intracavitary brachytherapy with sharp dose fall-off outside the target could be achieved with IMRT. The integral dose to planning target volume was significantly higher with HDR_BT in comparison with IMRT. Significant differences between the two techniques were seen for doses to 1 cc and 2 cc of rectum, while the differences in 1 cc and 2 cc doses to bladder were not significant. The integral doses to the nontarget critical and normal structures were smaller with HDR_BT and with IMRT. It is concluded that IMRT can be the choice of treatment in case of non-availability of HDR brachytherapy facilities or when noninvasive treatments are preferred

## Introduction

Brachytherapy is commonly used in the treatment of early or locally advanced cervical cancer. High dose rate intracavitary brachytherapy (HDR_BT) is a precise hypofractionated radiation treatment whose efficacy is well established in the treatment of cervical cancer. The amount of external-beam radiation that can be safely delivered to a big volume is often much less than the dose that is required to kill the tumor.[1] The pattern of the radiation dosage from intracavitary applicators allows the delivery of additional radiation to the volume of interest without exposing the surrounding normal tissues to excessive radiation. The placement of applicators within the patient aids in proper localization of the target and immobilization of the surrounding normal structures. Also brachytherapy avoids much of the geometric uncertainty present in the external-beam delivery techniques.

With the advent of intensity-modulated radiation therapy (IMRT), it is now possible for external-beam therapy to deliver complex dose distributions that conform to target volumes of arbitrary shape with rapid dose fall-off outside the target volume comparable to that achieved by brachytherapy. Few treatment planning studies comparing IMRT and HDR brachytherapy have been recently reported for cervical cancer,[2–4] prostate cancer[5] and endometrial cancer.[6] With the technique of conventional high dose rate brachytherapy with concomitant complementary IMRT boost, it is dosimetrically feasible to improve cervical tumor dose coverage.[7] Also the technical aspects of IMRT applied as a concomitant integrated boost for locally-advanced cervical cancer are discussed.[8] High dose to the target volume with good homogeneity can be safely achieved with IMRT. It has been shown that IMRT may permit escalation of the dose that can be safely delivered to the central pelvis and pelvic lymph nodes in post-hysterectomy cervical carcinoma.[9] Also it is concluded[10] that with IMRT, simultaneous integrated boost to replace the conventional two-phase treatments (whole pelvic irradiation followed by brachytherapy or external beam radiotherapy (EBRT) boost) is radiobiologically and dosimetrically feasible for locally advanced gynecological cancers that cannot be treated with brachytherapy for anatomical or medical reasons.

In the present study, an attempt has been made to investigate whether IMRT can achieve the pear-shaped dose distribution, which is characteristic of intracavitary brachytherapy with sharp dose fall-off outside the target and sparing of critical structures.

## Materials and Methods

Computed tomography scan images of 10 patients with tandem and ovoid applicator already treated with high dose rate intracavitary brachytherapy were used for this study. Initially the CT scan images were transferred to SomaVision workstation via Dicom RT. After reconstruction, the contouring was performed, and the images with the structure sets were transferred for IMRT and HDR_BT treatment planning. Brachytherapy treatment planning was performed in BrachyVision treatment planning system (Varian Medical Systems, Inc., Palo Alto, CA), and IMRT planning was performed using Eclipse treatment planning system (Varian Medical Systems, Inc., Palo Alto, CA) with Helios dose-volume optimizer. A conventional HDR brachytherapy plan was generated with a prescribed dose of 6 Gy in 5 fractions to point A. Point A was defined as per the Manchester system (2 cm superior along the tandem from the external cervical OS and 2 cm lateral to the intrauterine canal). A 5-mm step size was used. Dose optimization was performed by dragging the isodose lines using isodose reshape tool in order to reduce the critical organ doses. The 200%, 150%, 100%, 80% and 50% isodose lines were converted to structures and transferred to the Eclipse treatment planning system.

The IMRT plans were generated with a total prescription dose of 30 Gy in 5 fractions using sliding-window and step-and-shoot techniques.[11,12] For IMRT planning, the structure representing 100% isodose curve from brachytherapy treatment plan was defined as the point A isodose target volume. A sliding-window IMRT plan (IMRT_SW) was generated using 6-MV x-rays and 7 fields with equal spacing of the gantry angles (0°, 51°, 102°, 153°, 204°, 255°, 306°). Dose constraints were defined to cover 100% of the target volume with the prescription dose, while the maximum dose limit was 200% of the prescribed dose. Also dose constraints were set to the critical structures to keep their doses as low as possible. The same procedure was repeated for step-and-shoot IMRT planning. For step-and-shoot planning, after optimization the leaf motion calculations were performed for 3 intensity levels for each IMRT field, namely, 5 levels (Static_5), 10 levels (Static_10) and 20 levels (Static_20). The plans were normalized to obtain target coverage equal to that of the HDR_BT plan.

The dose distributions from HDR_BT and IMRT plans were compared visually on the axial, sagittal and coronal planes for degree of conformity of the prescribed dose to the planning target volume (PTV) and for inclusion of organs at risk (OARs) within high-dose and low-dose levels. Cumulative dose volume histograms (CDVHs) are recommended for evaluation of the complex dose heterogeneity. Also dosimetric parameters are developed and validated from dosimetric and clinical experiences at different institutions.[13] In the present study, CDVHs of target, bladder, rectum and normal tissue were analyzed, and certain dosimetric indices were evaluated. For target, dose to 95% of the target volume (D_95_), mean dose (D_mean_), conformity index (CI)[14] and homogeneity index (HI)[15] were used for comparison. The D_95_signifies relevant information about the high-dose regions within the target volume. Due to the steep dose gradient, small spikes in the contour cause large deviations in D_100._ Also D_95_ is less sensitive to these influences and hence it is more stable.[13] As the mean dose is representative of the average dose delivered within the target, D_mean_ is used for comparison. For OARs, dose to 5-cc volume (D_5cc_), dose to 2-cc volume (D_2cc_) and dose to 1-cc volume (D _1cc_) were compared. As there is rapid dose fall-off near the sources, in adjacent small contiguous organ volumes, the dose range needs to be indicated with ‘1cc’ and ‘2cc.’[13] For normal tissue, volume receiving 10% of prescribed dose (V_10_) was evaluated. The integral dose[16,17] [in joules (J)] was determined for the target and the nontarget structures, namely, bladder excluding PTV (Bladder_PTV); rectum excluding PTV (Rectum_PTV); and normal tissue volume, which is the body excluding PTV (Body_PTV).

The conformity[14] and homogeneity[15] indices were calculated as follows:

$$\mathrm{CI}\;=\;V_{REF}/V_{TOT’}$$

where V_REF_ represents the target volume encompassed by the reference isodose line, and V_TOT_ represents the total volume of the target.

$$\mathrm{HI}\;=\;\left(D_{5}-D_{95}\right)/D_{P’}$$

where D_5_ is the dose received by 5% of the target volume, D_95_ is the dose received by 95% of the target volume and D_P_ represents the prescribed dose to the target.

### Statistical analysis

Analysis was performed using a paired two-tailed Student t test to determine if there was a significant difference in any of the parameters analyzed. The differences were considered to be statistically significant at *P*-value ≥.05.

## Results and Discussion

**Figure 1:**
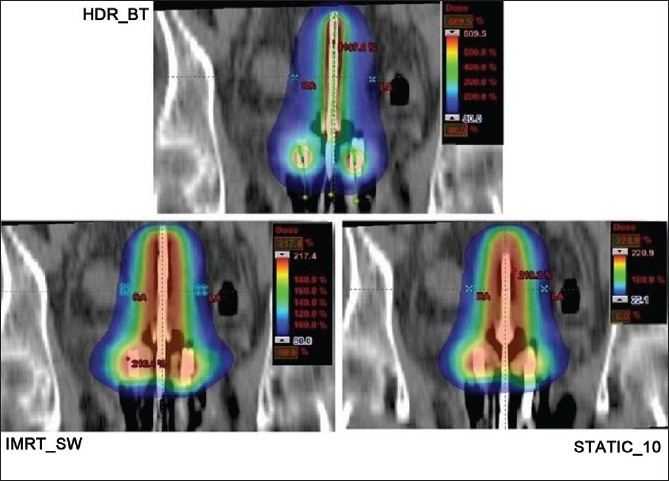
Coronal plane dose distributions of HDR_BT, IMRT and STATIC_10 plans

**Figure 2:**
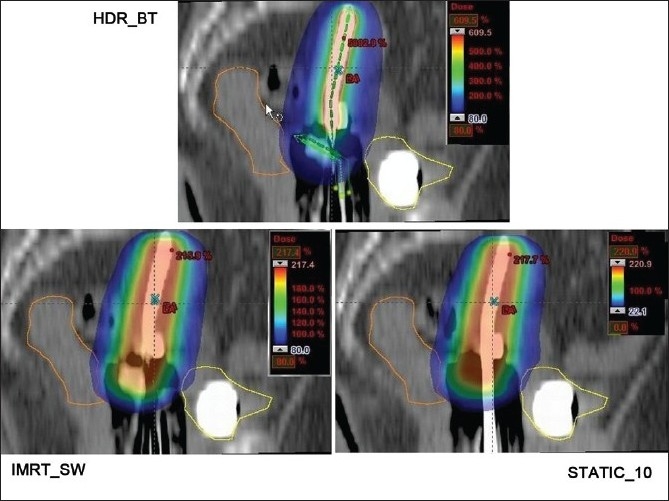
Sagittal plane dose distributions of HDR_BT, IMRT and STATIC_10 plans

**Figure 3:**
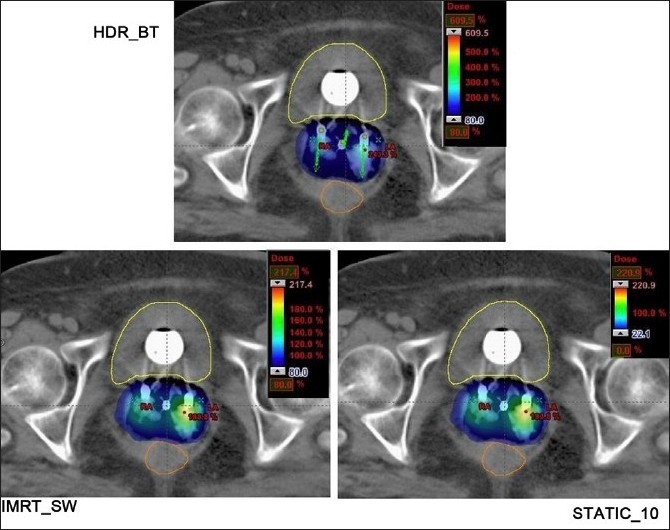
Axial plane dose distributions of HDR_BT, IMRT and STATIC_10 plans

**Figure 4:**
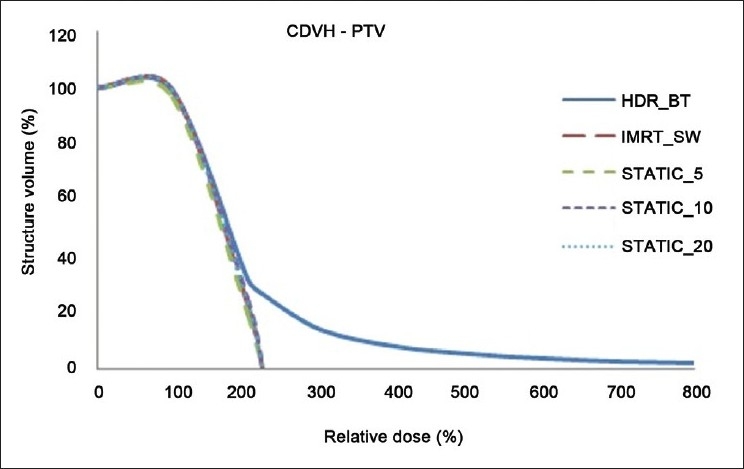
Cumulative DVHs of target for HDR_BT and IMRT plans

**Figure 5:**
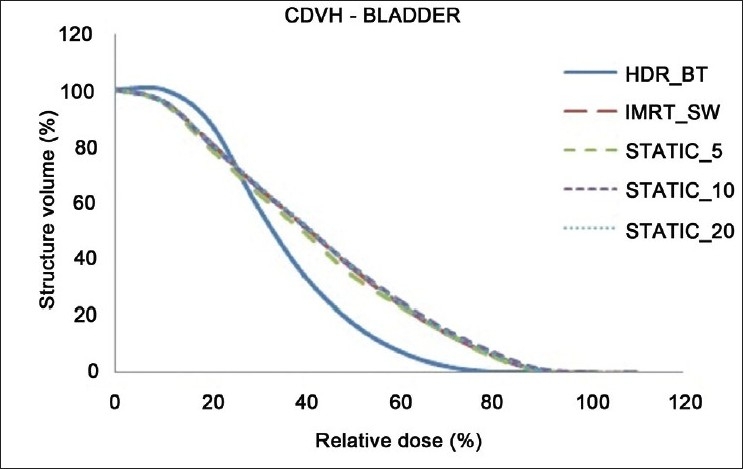
Cumulative DVHs of bladder for HDR_BT and IMRT plans

**Figure 6:**
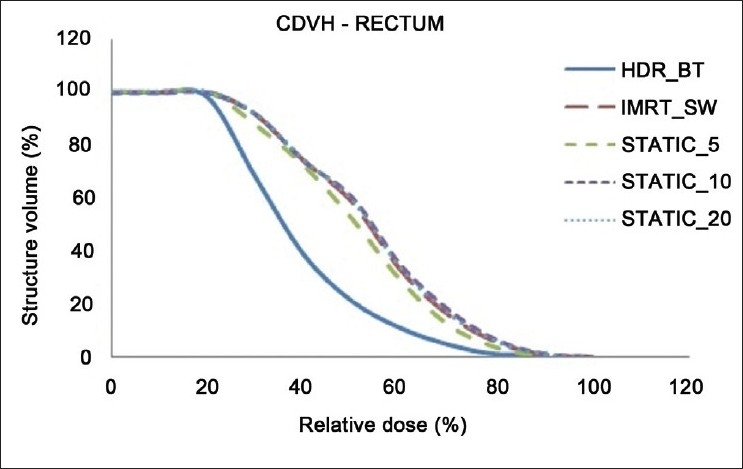
Cumulative DVHs of rectum for HDR_BT and IMRT plans

**Figure 7:**
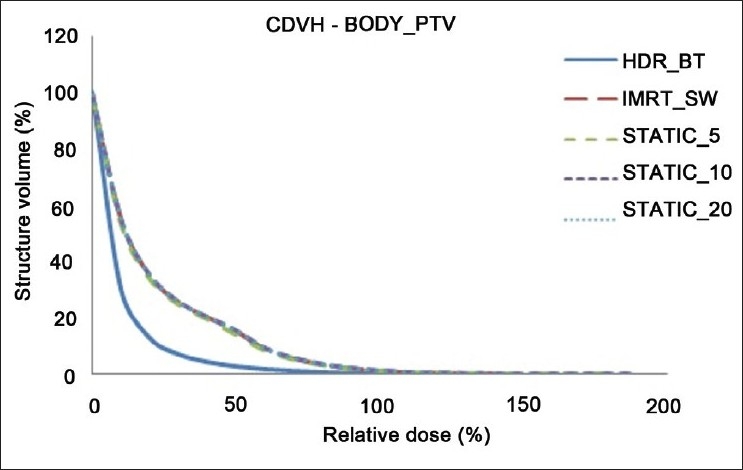
Cumulative DVHs of Body_PTV for HDR_BT and IMRT plans

The dose distributions in sagittal, coronal and axial planes obtained from HDR_BT, IMRT_SW and STATIC_10 techniques are shown in Figures 1–3, respectively. The cumulative DVHs of the target, bladder, rectum and normal tissue for the different plans are shown in Figures 4–7, respectively. The mean values of all the dosimetric parameters for 10 patients, along with the *P* values, are tabulated. The *P* values shown in Tables 1– 5 represent the difference between HDR_BT plan and each of the IMRT plans. Good target coverage was obtained with IMRT plans. The dose to 95% of the target volume was comparable between HDR_BT and IMRT, while the integral dose to PTV was significantly higher with HDR_BT as compared to IMRT.Table 2 shows the conformity and homogeneity indices. A CI value of unity is considered ideal for any treatment plan. Though the difference in the target conformity between HDR_BT and the IMRT techniques was statistically significant, ‘above 97%’ target coverage was achieved with all IMRT plans. Also smaller the value of HI, the better was the homogeneity in the target. Since the dose gradient within the target is very high in brachytherapy, it results in a higher HI value as compared to IMRT.

**Table 1:** Dosimetric parameters for the target volume with HDR-BT, IMRT_SW (and intensity-modulated radiation therapy), STATIC_5, STATIC_10 and STATIC_20 techniques

*Parameter*	*Mean ± Stdev for 10 patients (%)*				
	*HDR_BT*	*IMRT_SW*	*STATIC_5*	*STATIC_10*	*STATIC_20*
D_95_	103.74 ± 0.17	104.63 ± 3.83	104.69 ± 4.49	105.43 ± 3.62	105.03 ± 4.15
*P* value	—	0.491	0.522	0.182	0.359
D_MEAN_	217.21 ± 3.33	157.45 ± 8.33	162.21 ± 8.10	159.23 ± 7.96	158.20 ± 9.0
*P* value	—	0.0001*	0.0001*	0.0001*	0.0001*

* Indicates difference is statistically significant

**Table 2:** Conformity index and homogeneity index for the target

*Parameter*	*Mean ± Stdev for 10 patients*				
	*HDR_BT*	*IMRT_SW*	*STATIC_5*	*STATIC_10*	*STATIC_20*
CI	1.00 ± 0	0.97 ± 0.04	0.97 ± 0.02	0.98 ± 0.01	0.98 ± 0.02
P value	—	0.0249*	0.0018*	0.0082*	0.01*
HI	4.18 ± 0.20	1.19 ± 0.14	1.29 ± 0.13	1.21 ± 0.13	1.19 ± 0.14
P value	—	0.0001*	0.0001*	0.0001*	0.0001*

* Indicates difference is statistically significant

**Table 3:** Dosimetric parameters for the bladder with HDR_BT and IMRT

*Parameter*	*Mean ± Stdev for 10 patients (%)*				
	*HDR_BT*	*IMRT_SW*	*STATIC_5*	*STATIC_10*	*STATIC_20*
D_1cc_	81.00 ± 23.29	88.68 ± 15.66	91.71 ± 17.08	90.03 ± 16.08	89.59 ± 16.09
*P* value	—	0.162	0.102	0.1189	0.1419
D_2cc_	—	0.059	0.054	0.057	0.057
D_5cc_	61.36 ± 15.21	74.97 ± 15.29	76.80 ±16.84	75.85 ± 15.67	75.58 ± 15.73
*P* value	—	0.0025*	0.0051*	0.0021*	0.003*

* Indicates difference is statistically significant

**Table 4:** Dosimetric parameters for the rectum with HDR_BT and IMRT

*Parameter*	*Mean ± Stdev for 10 patients (%)*				
	*HDR_BT*	*IMRT_SW*	*STATIC_5*	*STATIC_10*	*STATIC_20*
D_1cc_	77.43 ± 17.1	87.67 ± 12.64	89.58 ± 11.64	89.18 ± 13.37	88.75 ± 12.85
*P* value	—	0.0021*	0.005*	0.001*	0.0022*
D_2cc_	69.13 ± 12.16	81.36 ± 8.90	83.13 ± 8.71	82.74 ± 9.65	82.29 ± 9.21
*P* value	—	0.0006*	0.0017*	0.0003*	0.0007*
D_5cc_	56.60 ± 7.62	71.84 ± 6.65	73.09 ± 7.36	73.00 ±7.28	72.60 ± 7.04
*P* value	—	0.0001*	0.0003*	0.0001*	0.0002*

* Indicates difference is statistically significant

**Table 5:** Normal tissue volume (Body_PTV) receiving 10% of the prescription dose with HDR_BT and IMRT

*Parameter*	*Mean ± Stdev for 10 patients (%)*				
	*HDR_BT*	*IMRT_SW*	*STATIC_5*	*STATIC_10*	*STATIC_20*
V_10_	33.06 ± 3.15	38.83 ± 14.17	37.78 ± 13.80	38.67 ± 14.08	38.83 ± 14.15
*P* value	—	0.0006*	0.0008*	0.0006*	0.0006*

* Indicates difference is statistically significant

Tables 3 and 4 represent the dosimetric parameters for bladder and rectum, respectively, with HDR_BT and IMRT plans. The dose to 5 cc of bladder and rectum, respectively, was significantly lower with HDR_BT as compared to IMRT. Statistically significant differences between the two techniques were seen for doses to 1 cc and 2 cc of the volumes of rectum, while the difference in the doses to 1 cc and 2 cc of bladder between HDR_BT and IMRT plans was not statistically significant. It can be seen from Table 5 that the volume of the normal tissue receiving 10% of the prescription dose was lower with HDR_BT as compared to IMRT plans. The integral doses to the target and nontarget structures are tabulated in Table 6. The integral doses to the nontarget critical structures are smaller with HDR_BT as compared to IMRT (sliding window and static) due to the rapid fall-off in dose beyond point A.

**Table 6:** Integral doses to target and nontarget structures with different plans

	*HDR_BT*	*IMRT_SW*	*STATIC_5*	*STATIC_10*	*STATIC_20*
PTV (J)	7.07	5.26	5.43	5.33	5.28
					
Bladder_PTV (J)	0.61	0.74	0.75	0.75	0.75
Rectum_PTV (J)	0.48	0.72	0.73	0.72	0.72
Body_PTV (J)	27.42	43.70	43.68	43.78	43.75

* Indicates difference is statistically significant

Table 1 shows that the mean dose delivered to the target was higher with STATIC_5 IMRT by 2.94% when compared with that delivered with IMRT_SW. It is observed that STATIC_5 and STATIC_10 IMRT plans provide better conformity to the target with higher doses to bladder and rectum (D_1cc,_ D_2cc_ and D_5cc_) as compared to IMRT_SW. Also STATIC_5 was found to deliver higher integral dose to the target as compared to the other IMRT techniques. Apart from these differences, the sliding-window and static IMRT plans showed comparable results for the dosimetric parameters analyzed.

In this study, the feasibility of comparing the dose distribution of intracavitary brachytherapy with that of IMRT was studied. A dose fall-off up to 80% isodose line could be achieved with IMRT, which is comparable to that achieved with HDR_BT. It was observed that when IMRT optimization was performed to achieve the higher dose envelope from HDR_BT isodose distribution (150% and 200% isodose lines in the present case), the integral dose to the body and normal tissue increased considerably. However, the dose distribution was found to be more homogeneous with IMRT, as represented by the maximum target doses from both techniques in Figure 4.

The present study is a comparison of dose distribution to the target and neighboring critical structures with IMRT and HDR_BT. In practice, the effective total dose to be delivered to target with IMRT needs to be computed by accounting the equivalent biological effective doses (BEDs) for HDR_BT and IMRT. Also the early and late effects on normal structures need to be considered in determining the total dose to be delivered with IMRT.

The integral dose to PTV was significantly higher with HDR_BT due to the immensely high dose close to the applicators, while dose to the normal tissue was lower. On the other hand, IMRT delivers low doses to larger volumes of the normal tissue. This irradiation of normal tissue with low doses in IMRT is one of the causes of secondary malignancies.[18] Also the long latency period for radiation-induced tumors may result in radiation-related second malignancy risk becoming a more significant issue.[19] Moreover, in literature[2,3] it has been pointed out that in brachytherapy the extremely high doses close to the radioactive source may not provide improvement in tumor control.

A comparison of the 2D and 3D volumetric CT-based calculations has shown that the International Commission on Radiation Units and Measurements(ICRU) rectal point is a reasonable surrogate for the minimal dose to the most irradiated 2 cc of the rectal wall,[20,21] and this 2 cc volume was found to be a clinically relevant parameter correlating with complications.[22] But it was found that the ICRU bladder point is an uncertain predictor of the minimal dose to the most irradiated 2 cc of the bladder, as shown by earlier few studies.[20,21,23] Regarding the HDR_BT treatment, it is generally delivered with no rectal content and urine drained continuously from the patient’s bladder. Similar treatment condition may be simulated during IMRT by duly instructing the patient to empty the bladder and rectum prior to treatment. Hence the same treatment condition may be reproduced with the cooperation of the patient for brachytherapy and IMRT treatments. Setup uncertainties are another concern when treating with IMRT, which can be effectively overcome by the modern techniques of image guidance, by using proper immobilization devices and by using additional margins to the target volume. However, clinical evaluation of IMRT with dose distribution similar to that of intracavitary brachytherapy is necessary before implementation of the technique for patient treatment.

## Conclusion

It was seen that the pear-shaped dose distribution characteristic of intracavitary brachytherapy with dose fall-off outside the target could be achieved with IMRT at the level of point A. HDR_BT allows higher mean doses to be delivered to PTV with reduced critical-organ doses as compared to IMRT. Nevertheless, IMRT can be considered as an option to replace intracavitary brachytherapy in situations where HDR brachytherapy facilities are unavailable or when noninvasive treatment techniques are preferred.
